# Family history and stroke outcome in a bi-ethnic, population-based stroke surveillance study

**DOI:** 10.1186/1471-2377-5-20

**Published:** 2005-10-31

**Authors:** Lynda D Lisabeth, Melinda A Smith, Devin L Brown, Ken Uchino, Lewis B Morgenstern

**Affiliations:** 1 Stroke Program, University of Michigan Medical School, Ann Arbor, Michigan, USA; 2 Stroke Institute, University of Pittsburgh Medical Center, Pittsburgh, Pennsylvania, USA; 3 Department of Epidemiology, University of Michigan School of Public Health, Ann Arbor, Michigan, USA

## Abstract

**Background:**

The genetic epidemiology of ischemic stroke remains relatively unstudied, and information about the genetic epidemiology of ischemic stroke in populations with significant minority representation is currently unavailable.

**Methods:**

The Brain Attack Surveillance in Corpus Christi project (BASIC) is a population-based stroke surveillance study conducted in the bi-ethnic community of Nueces County, Texas, USA. Completed ischemic strokes were identified among patients 45 years or older seen at hospitals in the county between January 1, 2000 – December 31, 2002. A random sample of ischemic stroke patients underwent an in-person interview and detailed medical record abstraction (n = 400). Outcomes, including initial stroke severity (NIH stroke scale), age at stroke onset, 90-day mortality and functional outcome (modified Rankin scale ≥2), were studied for their association with family history of stroke among a first degree relative using multivariable logistic and linear regression. A chi-square test was used to test the association between family history of stroke and ischemic stroke subtype.

**Results:**

The study population was 53.0% Mexican American and 58.4% female. Median age was 73.2 years. Forty percent reported a family history of stroke among a first degree relative. Family history of stroke was borderline significantly associated with stroke subtype (*p *= 0.0563). Family history was associated with poor functional outcome in the multivariable model (OR = 1.87; 95% CI: 1.14–3.09). Family history was not significantly related to initial stroke severity, age at stroke onset, or 90-day mortality.

**Conclusion:**

Family history of stroke was related to ischemic stroke subtype and to functional status at discharge. More research is needed to understand whether stroke subtype would be a useful selection criterion for genetic association studies and to hypothesize about a possible genetic link to recovery following ischemic stroke.

## Background

Data from different sources, including animal and family history studies, suggest that genetic factors play a role in ischemic stroke [1-4]. Despite the challenges associated with studying the genetics of a heterogeneous disorder, several polymorphisms have been identified for ischemic stroke with small to modest effects [5]. Most recently, researchers using data from an Icelandic population have identified a locus on chromosome 5 thought to be associated with ischemic stroke in humans [6]. While the focus has been on identifying genetic determinants of stroke risk, many aspects of the genetic epidemiology of ischemic stroke remain relatively unstudied. Aside from the relationship between a positive family history of stroke and stroke risk, aspects of ischemic stroke for which genetic factors are plausible, such as stroke subtype, stroke severity and stroke outcomes, have not been thoroughly investigated.

Further, information about the genetic epidemiology of ischemic stroke in populations with significant representation from minority groups, such as Hispanic Americans, is currently unavailable. Differences in stroke risk, age at onset of stroke and family history of stroke between Hispanics and non-Hispanic whites raise the possibility of genetic differences by ethnicity [7,8]. The objective of this study was to provide an overview of the relationship between family history of stroke and the epidemiology of ischemic stroke including associations with stroke subtype, age at stroke onset, stroke severity, mortality, and functional status following stroke using data from a bi-ethnic study population of ischemic stroke cases aged greater than 44 years.

## Methods

BASIC is a population-based stroke surveillance study conducted in Nueces County, Texas, USA. Methods of the Brain Attack Surveillance in Corpus Christi (BASIC) Project have been reported [9]. Due to its distance from Houston and San Antonio, investigation in Nueces County allows for complete case capture for first medical contact in acute stroke. The population size of the county is 313,645, and 95% of the population resides within the city of Corpus Christi. Non Hispanic whites (NHW) comprise 38% of the population, and Mexican Americans (MA) comprise 56%.

Acute cerebrovascular events (completed ischemic strokes, transient ischemic attacks, intracerebral hemorrhages (ICH), and subarachnoid hemorrhages (SAH)) were identified among patients 45 years and over who were seen (including those seen in the emergency department and not admitted) at one of the seven area hospitals between January 1, 2000 through December 31, 2002 using active and passive surveillance methods. Cases that did not present to a hospital were identified through a simple random sample from 45 primary care physicians from the community and four nursing homes, and from all 11 neurologists practicing in the county. Cerebrovascular events were validated by fellowship-trained stroke neurologists based on published criteria and blinded to subjects' race-ethnicity and age [10]. Only ischemic strokes were included in this study. For those individuals with multiple ischemic strokes (n = 5) within the time period, only the first was considered for this analysis. Therefore, the final population for analysis incorporated incident strokes as well as recurrent strokes that occurred during the study time period, but was limited to one stroke per individual.

### Outcomes

For this analysis, five outcomes were studied for their possible association with family history of stroke as outlined in Table 1. How each outcome was measured and defined for this analysis is described below.

**Table 1:** Outcomes and end points investigated for possible associations with family history of stroke.

Outcome	End Point
Ischemic stroke subtype	TOAST criteria (large artery atherosclerosis, cardioembolism, small vessel occlusion, stroke of other determined etiology, and stroke of undetermined etiology) plus additional category of non-lacunar stroke of unknown etiology[13]
Age at stroke onset	Continuous age
Initial stroke severity (NIHSS)	Continuous NIHSS
Functional outcome (mRS)	mRS ≤1 vs mRS >1
Mortality	Death ≤ 90 days vs Alive at 90 days

NIHSS = NIH stroke scale, mRS = modified Rankin scale

### Interview methodology and family history data

A random sample comprising two-thirds of patients with validated cerebrovascular events was asked to participate in an in-person interview with the goal of achieving a 50% sample of stroke cases. The response rate for interview was 84% [11]. The interview contained questions regarding family history of stroke among first degree relatives (parents, siblings, and children). Patients unable to answer appropriately to a series of orientation questions asked before the interview had a proxy interview, in the presence of the patient whenever possible. Proxy interviews were conducted with the person who knew the patient's daily activities and medical history the most. A previous analysis revealed the agreement between patient/proxy interviews for six critical elements (insurance status, use of a routine physician, history of hypertension, history of diabetes, current smoking status, educational level, and trust in doctors and nurses) ranged from 84% to 100%. Interviews were performed in English or Spanish depending on the patients' language preferences.

A random sample of the patients interviewed was selected to undergo a more detailed medical record abstraction, including reports of medical testing performed and information necessary to classify ischemic strokes according to subtype. The random sample with both interview data and detailed medical record abstraction was used for this analysis.

### Ascertainment of outcomes

Ischemic stroke cases were classified by two fellowship-trained stroke neurologists into five stroke subtype categories according to the criteria of the Trial of ORG 10172 in Acute Stroke Treatment (TOAST) Study: large artery atherosclerosis, cardioembolism, small vessel occlusion, stroke of other determined etiology, and stroke of undetermined etiology [12]. An additional category of non-lacunar stroke of unknown etiology was developed that comprised large strokes that had insufficient evidence for categorization into large artery atherosclerosis or cardioembolism [13]. The inter-rater agreement between the two neurologists for determination of subtype was high (kappa 0.80, p < 0.001). Neurologists were blinded to the subject's ethnicity and age.

Initial stroke severity was measured using the NIH stroke scale (NIHSS). The NIHSS was retrospectively abstracted from the chart in accordance with the validated method of Williams et al [14]. Functional outcome was assessed at discharge using a modified Rankin scale (mRS) calculated by the abstractors using data in the medical record. For the purposes of this analysis, the mRS, which ranges from 0–6, was dichotomized to represent good (0–1) and poor (2–6) functional outcome. This categorization of the mRS has been shown to be more powerful than other choices for cut points for this scale [15].

Deaths among the stroke cases were identified for the time period January 1, 2000 through December 31, 2003 by four methods: 1) surveillance of hospitalized or emergency department stroke cases, 2) Texas Department of Health (TDH), 3) Social Security Death Index (SSDI), and 4) Nueces County coroner. If a stroke case died in-hospital, the death was recorded by the abstractors during surveillance. Otherwise, deaths were captured using methods 2–4 above. TDH provided death certificates for Texas residents electronically, with a one-year lag period to ensure complete capture. Demographic data from the medical record, including first name, last name, social security number, date of birth, and permanent address, was crossed-referenced with the TDH death certificate database. At least three of the five items must have been identical for the BASIC stroke case to be considered a match with the mortality case from TDH. In an attempt to capture deaths that occurred outside of Texas and not captured by TDH, we linked cases not identified as having died using the first two data sources to the SSDI. We also routinely screened records from the coroner. All deaths reported to the coroner have a death certificate filed with TDH.

### Statistical analysis

Frequencies and percents were calculated for categorical variables. Means and medians were calculated for continuous variables. A chi-square test was used to test the association between family history of stroke among a first degree relative and ischemic stroke subtype. Multivariable logistic regression was used to test the association between a positive family history of stroke among a first degree relative and functional outcome (dichotomized mRS) and 90-day mortality. Covariates were selected for inclusion in the models in a pre-specified fashion based on their plausible relationship with the given outcome or their ability to confound family history. In each logistic model, the number of covariates was limited based on the rule of 10 which requires at least 10 least frequent outcomes for each degree of freedom [16]. For the model predicting functional outcome the following covariates were included: age, gender, ethnicity, hypertension, diabetes, atrial fibrillation, coronary heart disease, NIHSS, and stroke subtype. For the model predicting 90-day mortality, the following covariates were included: age, gender, ethnicity, and NIHSS.

Multivariable linear regression was used to test the association between a positive family history of stroke among a first degree relative and initial stroke severity (NIHSS) and age at stroke onset. Again, covariates were selected for inclusion in the models in a pre-specified fashion. For the models predicting NIHSS and age at stroke onset the following covariates were included: gender, ethnicity, hypertension, diabetes, atrial fibrillation, coronary heart disease, high cholesterol, smoking, and stroke subtype. Age was also included in the model for NIHSS.

Family history was modeled dichotomously in all models (yes/no). Ethnicity (MA vs. NHW), gender (female vs. male) and the history variables (smoking, high cholesterol, coronary artery disease, diabetes, atrial fibrillation, hypertension) were treated dichotomously. Age was treated continuously. Stroke subtype was modeled as a series of indicator variables with cardioembolic strokes as the referent.

Chi-square tests were used to test the association between use of proxy subjects and family history and to compare the demographic characteristics of cases with and without proxies. To assess whether the use of proxy subjects confounded the relationship between family history and the five outcomes, a covariate representing proxy subject (yes/no) was also added to each of the multivariable models and the degree to which the point or parameter estimate changed was evaluated. A greater than 10% change was considered to represent confounding. A chi-square test was used to test the association between use of proxy subjects and stroke subtype.

The project was approved by the Institutional Review Boards at the University of Texas, Houston and University of Michigan, and each Nueces County hospital.

## Results

There were 2,550 validated cerebrovascular events between January 1, 2000 and December 31, 2002. Ischemic strokes constituted 1,477 of the 2,550 events. One-hundred six cases who were not MA or NHW (7 Asian, 89 African American, 10 unknown race/ethnicity) were excluded due to small sample size. Eight-hundred seventeen of these cases were interviewed and 405 of the cases were subtyped based on documentation gathered during the extended medical record abstraction. Five recurrent events were eliminated resulting in 400 ischemic stroke cases for the final analysis.

Of the 400 cases interviewed, 39.5% reported a positive family history of stroke among a first degree relative. Eleven percent (n = 44) were unsure of a family history of stroke and for three cases (0.8%), this question was not answered. Table 2 displays baseline characteristics for stroke subjects with complete family history data (n = 353). The study population was 53.0% (n = 187) MA and 58.4% (n = 206) female. Median age was 73.2 years (IQR: 65.2–79.7). There were no differences in the prevalence of stroke risk factors among those with and without a positive family history of stroke. Women were more likely to report a family history of stroke compared with men as we have previously reported [8].

**Table 2:** Demographics and risk factors for ischemic stroke cases with complete family history data (n = 353).

	Family History				
	
	Yes (n = 158)		No (n = 195)		
			
	N	%	n	%	p-value
MA	90	57.0	97	49.7	0.1773
NHW	68	43.0	98	50.3	
					
Females	108	68.4	98	50.3	0.0006
Males	50	31.7	97	49.7	
					
Risk Factors					
Hypertension	120	76.0	138	70.8	0.2759
Diabetes	76	48.1	80	41.0	0.1838
Atrial Fibrillation	18	11.4	26	13.3	0.5836
Coronary Heart Disease	51	32.3	71	36.4	0.4423
Hyperlipidemia	25	15.8	44	22.6	0.1128
Smoking	40	25.3	60	30.8	0.2589
History of Stroke/TIA	62	39.2	66	33.9	0.2952

MA = Mexican American, NHW = non-Hispanic white

The distribution of ischemic stroke subtype among the 400 cases was 21.0% cardioembolic, 14.3% large vessel, 19.3% small vessel, 22.8% large strokes with insufficient evidence for categorization into large artery atherosclerosis or cardioembolism, 21.5% undetermined etiology, and 1.3% other determined etiology. Family history of stroke was borderline significantly associated with ischemic stroke subtype (*p *= 0.0563), with family history less frequent among those with large vessel strokes and more frequent among those with small vessel disease (Table 3).

**Table 3:** Distribution of ischemic stroke subtypes by family history (n = 353).

	Family History			
	
Ischemic Stroke Subtype	Yes		No	
		
	n	%	n	%
Cardioembolic	37	23.4	32	16.4
Large vessel	16	10.1	35	18.0
Non-lacunar/large stroke*	34	21.5	44	22.6
Small vessel	39	24.7	33	16.9
Undetermined	29	18.4	49	25.1
Other	3	1.9	2	1.0

Total	158	100.0	195	100.0

χ^2 ^(df 5) = 10.76, p = 0.0563				

* Non-lacunar strokes of unknown etiology comprised of large strokes with insufficient evidence for categorization into large artery atherosclerosis or cardioembolism

Results from the multivariable logistic regression models are displayed in Table 4. Among the 400 cases, 16.0% died within 90 days. Among those with a positive family history, 16.5% died within 90 days, and among those with no history, 12.3% died. Family history showed a positive association with 90-day mortality (OR = 1.55, 95% CI: 0.77–3.13), although this association did not reach significance in the multivariable model.

**Table 4:** Association of family history of stroke with functional outcome and mortality (n = 353).

End Point	OR	95% CI
Rankin ≥ 2^1^	1.87	(1.14, 3.09)
		
90-day Mortality^2^	1.55	(0.77, 3.13)

OR = odds ratio, CI = confidence interval

1 Adjusted for age, gender, ethnicity, hypertension, diabetes, atrial fibrillation, coronary artery disease, initial stroke severity, ischemic stroke subtype

2 Adjusted for age, gender, ethnicity, initial stroke severity

Among the 353 cases with family history data, 61.5% had a mRS ≥ 2. Sixty-nine percent of those with a positive family history had poor functional outcome compared to 55.4% in those with no family history. Family history was positively associated with poor functional outcome in the multivariable model (OR = 1.87; 95% CI: 1.14–3.09) adjusted for the other covariates.

Results from the multivariable linear regression models are displayed in Table 5. Median age at stroke onset was 71.4 years among those with a family history and 73.9 years among those with no family history. Family history was associated with a younger age at stroke onset in the multivariable model (β = -1.38, standard error = 1.18), but this relationship was not statistically significant. Median NIHSS was 4 among those with a family history and 3 among those with no family history. Results from the multivariable model (Table 5) suggest a mean NIHSS difference of 0.41 between those with and without a family history, but this association did not reach significance.

**Table 5:** Association of family history of stroke with initial stroke severity and age at stroke onset (n = 353).

End Point	β	Std Error
NIH stroke scale^1^	0.41	0.72
		
Age at stroke onset^2^	-1.38	1.18

Std Error = standard error

1 Adjusted for age, gender, ethnicity, hypertension, diabetes, atrial fibrillation, coronary artery disease, high cholesterol, smoking, ischemic stroke subtype

2 Adjusted for gender, ethnicity, hypertension, diabetes, atrial fibrillation, coronary artery disease, high cholesterol, smoking, ischemic stroke subtype

### Use of proxies

Thirty-seven percent (n = 147) of the interviews were conducted with proxies. In 80.7%, the proxy subject was a spouse (31.7%) or child (49.0%) of the patient. The use of proxies varied with age of the stroke case (*p *< 0.0001). Median age of cases with a proxy interview was 78.8 years and median age of cases without a proxy interview was 69.6 years. Use of proxies did not differ by gender or ethnicity of the case.

Family history did not vary by use of proxy subjects (*p *= 0.7560). However, the use of a proxy did appear to confound the relationship between family history and age at stroke onset as evidenced by the change in the parameter estimate with inclusion of this covariate (Table 6). Family history remained insignificant (*p *= 0.2817) in this model regardless of inclusion of the variable for proxy subject. Use of proxy subjects was significantly related to stroke subtype (*p *< 0.0001).

**Table 6:** Comparison of point/parameter estimates for family history before and after adjustment for use of proxy subjects (n = 353).

	Original Model	Model with Proxy
	
End Point	OR/Parameter Estimate for Family History	OR/Parameter Estimate for Family History
Rankin ≥ 2	1.87	1.90
90-day Mortality	1.55	1.49
NIH stroke scale	0.41	0.42
Age at stroke onset	-1.38	-1.19

## Discussion

In our study population of ischemic stroke cases, family history of stroke among a first degree relative was associated with poor functional outcome, defined as a modified Rankin scale score ≥ 2 at discharge, adjusted for initial stroke severity and other potential confounders. The associations of family history with initial stroke severity and 90-day mortality, although not significant, together with the association with poor functional outcome at hospital discharge, suggested family history was related to more severe strokes among the cases. Initial stroke severity and functional outcome are known to be highly correlated and were significantly associated in our data [17,18]. Possible explanations for the lack of a significant association of family history with initial stroke severity include reduced power to detect a difference due to our sample size and possible clustering or downward bias of NIHSS score assignments due to the retrospective designation of these scores. Familial factors that influence poor short-term recovery after stroke could explain the finding of a positive association of family history with poor functional outcome at discharge and its lack of association with initial NIHSS. Additional large-scale studies are needed to confirm our findings and to suggest a potential genetic link with recovery after stroke.

There may be differences in genetic susceptibility to stroke based on ischemic stroke subtype. We found a borderline significant association with the distribution of subtype and family history, with the data suggesting that cases with small vessel disease were more likely to have a family history of stroke. This result is consistent with results from hospital-based studies of stroke cases and controls [19-21]. Our data also suggested that cases with large vessel disease were less likely to have a family history. Previous studies have found a greater prevalence of family history among those with large vessel disease [19-21]. In our study population, large vessel disease was present in 14.3% of cases. This percent is less than other study populations and perhaps contributes to our different findings. We also had 21.5% of cases with undetermined etiology possibly due to less extensive evaluation documentation in community hospitals. There may also be a genetic susceptibility to the same stroke type in the case as that of the family member [19], but we were unable to explore this question as the stroke type of the family member is unknown. First degree relatives with stroke who are parents of the case are often deceased by the time of the offspring's stroke, adding to the challenge of classifying the family members' stroke type.

Previous studies have found associations between family history of stroke and earlier age at stroke onset [20,22] suggesting age may be a useful selection criteria for future ischemic stroke genetics studies. Our findings with regard to age at stroke onset were in this direction but not significant after multivariable adjustment for confounders and independent predictors.

Strengths of the current study include: 1) the population-based design and ascertainment of all cases presenting for medical attention to a hospital, including those seen in the emergency department but not subsequently hospitalized, 2) the inclusion of ischemic strokes only, as not all types of stroke are equally likely to be heritable, 3) the detailed subtyping of ischemic strokes to allow for comparison of family history across subtypes, and 4) the inclusion of a significant proportion of Mexican Americans, a minority population with increased risk of stroke. Further, we adjusted for potential confounding factors including stroke risk factors, stroke severity, demographics and stroke subtype when sample size and the frequency of the outcome permitted.

Some limitations of this study warrant discussion. Family history data was based on self-report by stroke cases and was not validated through source documentation or interviews with family members. We interviewed a proxy subject in lieu of a patient interview when the patient could not answer a series of orientation questions. In the majority of these cases (84%), the interview was with a spouse, sibling, or child who would likely have knowledge of the family history. Further, the use of proxy subjects did not appear to confound the relationship between family history and the outcomes with the exception of age at stroke onset; however, family history remained an insignificant predictor of age at stroke regardless of proxy use. Some stroke cases were unaware of their family history (11%), and this could have biased our findings regarding family history; however, the effect is likely to be small. We did not have data on family size, which may impact family history of stroke, and were unable to account for this factor in our analysis. We also did not have information on age of the first-degree relative at the time of the stroke. Stroke subtypes were classified using data from abstracted medical charts including radiology reports so misclassification of subtype may have occurred because of lack of full information. Furthermore, the TOAST criteria may not be an accurate reflection of stroke mechanism; although a superior classification system is not available [23]. The mRS was retrospectively assessed at discharge by data abstractors, a method that has not been previously validated, but all abstractors were trained by the same individual with training reinforced by a study neurologist. The BASIC study population is over 50% Mexican American and thus differs from the general US population. Findings from this study population may not be generalizable. Several end points were assessed in our analysis and thus readers are cautioned regarding the multiple comparisons, although the number of comparisons was not large at five.

## Conclusion

In our study population, we found that a positive family history of stroke among a first degree relative was related to ischemic stroke subtype and also to functional status at discharge. More research is needed to understand whether stroke subtype would be a useful selection criterion for genetic association studies and to hypothesize about a possible genetic link to recovery following ischemic stroke.

## Competing interests

The author(s) declare that they have no competing interests.

## Authors' contributions

LDL conceived of this analysis from the BASIC study, performed statistical analysis, and drafted the manuscript. MAS prepared the data for analysis and critically reviewed the manuscript. DLB helped to interpret the data analysis and draft the manuscript. KU carried out the ischemic stroke subtyping and critically reviewed the manuscript. LBM is the principal investigator of BASIC. He helped to conceive of this analysis, performed ischemic stroke validation and subtyping, helped interpret the data analysis, and critically reviewed the manuscript. All authors read and approved the final manuscript.

## Pre-publication history

The pre-publication history for this paper can be accessed here:


